# Two novel mutations in *DNAJC12* identified by whole‐exome sequencing in a patient with mild hyperphenylalaninemia

**DOI:** 10.1002/mgg3.1303

**Published:** 2020-06-10

**Authors:** Mengting Li, Qi Yang, Sheng Yi, Zailong Qin, Jingsi Luo, Xin Fan

**Affiliations:** ^1^ Department of Genetic and Metabolic Central Laboratory Maternal and Child Health Hospital of Guangxi Zhuang Autonomous Region Nanning China; ^2^ Department of Pediatric The Second Affiliated Hospital of Guangxi Medical University Nanning China

**Keywords:** *DNAJC12*, hyperphenylalaninemia, whole‐exome sequencing

## Abstract

**Background:**

Recently hyperphenylalaninemia (HPA) caused by variants in *DNAJC12* was reported and this suggested a new strategy for diagnosis. But *DNAJC12*‐associated HPA is a rare in Chinese population so far.

**Methods:**

The clinical information and blood samples from the patient and his family members were collected and analyzed. Whole‐exome sequencing (WES) was used to identify the causative gene.

**Results:**

We reported a newborn patient with HPA, having excluded the causes in common genes associated with HPA. By using whole‐exome sequencing, novel compound heterozygosity mutations in *DNAJC12* were found, namely c.306C>G (p.His102Gln) and c.182delA (p.Lys61Argfs*6). Administering a diet with low phenylalanine combined with tetrahydrobiopterin and neurotransmitter precursors were shown to be effective in preventing neurodevelopmental delay for these patients.

**Conclusion:**

Our finding confirms the diagnosis of *DNAJC12*‐associated HPA and suggests that genetic detection of *DNAJC12* should be considered when newborn screening results are positive for HPA.

## Graphical abstract

By using whole‐exome sequencing, we found novel compound heterozygosity mutations in DNAJC12, namely c.306C>G (p.His102Gln) and c.182delA (p.Lys61Argfs*6), which were considered to be the genetic cause of a patient with hyperphenylalaninemia.

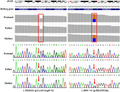

## 1 INTRODUCTION

Hyperphenylalaninemia (HPA) is a common congenital disorder of amino acid metabolism, where the patient is unable to catalyze the hydroxylation of phenylalanine to tyrosine in the normal way. Such patients usually have variants in *PAH* (MIM: 612349) or *PTS* (MIM: 612719), which are the most common causes of HPA (Blau, 2016). Recently, HPA due to variants in a new gene, *DNAJC12* (MIM: 606060), was reported. *DNAJC12*, also known as JDP1 or HPANBH4, is a member of the DnaJ/Hsp40 family which acts as a co‐chaperone to regulate protein folding, transport, translational initiation, and gene expression (Lee, Hahn, Yun, Mita, & Chung, 2000). Most lines of research into *DNAJC12* are focused on cancer and Parkinson's disease (De Bessa et al., 2006; Feng et al., 2019). The diagnosis of *DNAJC12*‐associated HPA was reported for the first time in 2017 (Anikster et al., 2017). Subsequently, there were several reports of pathogenic mutations of *DNAJC12* associated with HPA (Blau, Martinez, Hoffmann, & Thony, 2018; Veenma, Cordeiro, Sondheimer, & Mercimek‐Andrews, 2018).

In the present study, we report the identification of novel compound heterozygosity mutations in *DNAJC12* from a proband with HPA and no reported mutation was found in either *PAH* or *PTS*. In addition, this is the first report of *DNAJC12*‐associated HPA in Guangxi Zhuang Autonomous Region, China.

## 2 SUBJECTS AND METHODS

### 2.1 Ethics approval

All procedures in this study were approved by the Institutional Review Boards and Ethics Committees of Maternal and Child Health Hospital of Guangxi Zhuang Autonomous Region. Informed consent was obtained from participants in the study.

### 2.2 Clinical data collection

The patient was from Maternal and Child Health Hospital of Guangxi Zhuang Autonomous Region. The clinical information and blood samples from the patient and his family members were collected and analyzed. The blood concentrations of phenylalanine and tyrosine were analyzed by tandem mass spectrometry or fluorescence detection. Urinary pterin analysis including the urinary concentrations of neopterin and biopterin were analyzed by high‐performance liquid chromatography (HPLC). Dihydropteridine reductase (DHPR) activity was detected in dried blood spots.

### 2.3 Molecular analysis

Genomic DNA was extracted from peripheral whole blood using the nucleic acid extraction system (Zeesan Biotech, China). Whole‐exome sequencing (WES) for the patient and his family members was performed using Hiseq2000 (Illumina, San Diego, America). The TGex software (LifeMap Sciences, Alameda, CA) was used to annotate the selected variants which were confirmed by Sanger sequencing. The primers of *DNAJC12* (NM_021800.2) were shown in Table S1. Rare variants were evaluated and classified following the ACMG/AMP standards and guidelines (Richards et al., 2015).

## 3 RESULTS

### 3.1 Clinical characteristic of the patient

The proband was a full‐term birth boy with normal height and weight. He was first found to be affected by HPA during newborn screening (NBS) and this was confirmed by further tests. Urinary concentrations of neopterin or biopterin and dried blood spot DHPR activity were normal. Detailed clinical information of patient is available in Table 1.

**TABLE 1:** Clinical information of the proband

Clinical information	Proband	Normal reference values
Sex	male	‐
Phe (mg/dl) ^1^	3.72 ↑	<2.0
Phe (μ mol/L) ^2^	223.14 ↑	25–120
Phe/Tyr	4.94 ↑	0.2–2.0
DHPR activity (nmol/min./5 mm disc) ^3^	2.17	1.02–3.35
Neopterin (*N*, mmol/mol Cr) ^4^	0.885	1.2–2.92
Biopterin (B, mmol/mol Cr) ^4^	0.64	0.42–1.92
B% (B/(*N* + B)%)	42.28	19.8–50.3

Note

Phe: phenylalanine; Tyr: tyrosine; Method: 1: time‐resolved fluorescence; 2: tandem mass spectrometry; 3: UV‐2450 double‐beam spectrophotometer; 4: high‐performance liquid chromatography

### 3.2 Whole‐exome sequencing diagnosis of *DNAJC12* deficiency

**FIGURE 1:**
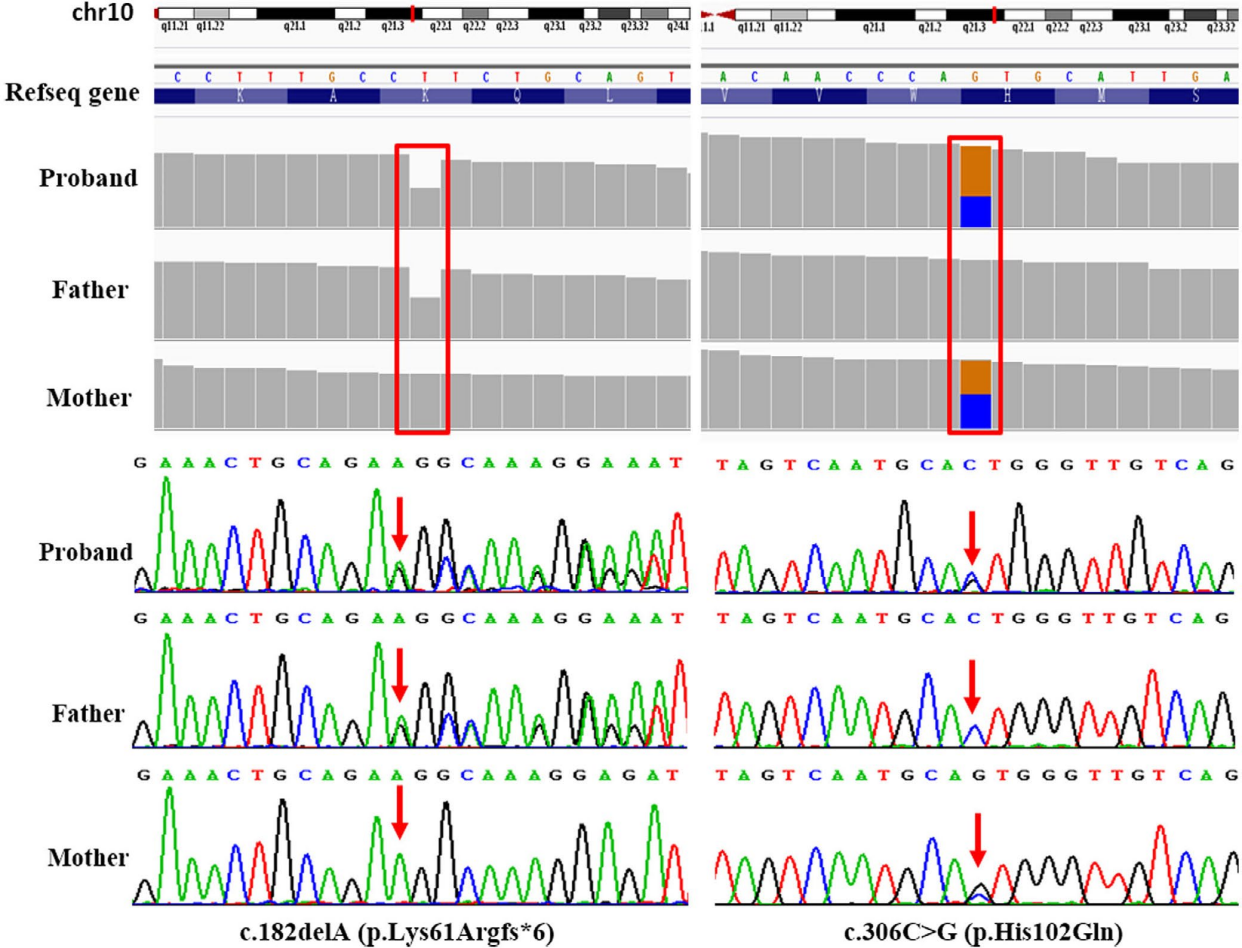
Whole‐exome sequencing diagnosis of family members with mutations in *DNAJC12*. Whole‐exome sequencing results showed mutations in *DNAJC12* (NM_021800.2) were inherited from each parent and this was confirmed by Sanger sequencing

The patient first accepted Sanger sequencing of *PAH* and *PTS*, but there was no reported pathogenic variation detected. When he was a 1‐year‐old infant, he underwent WES to confirm the genetic cause of HPA. No mutations in the *PAH*, *PTS,* and other genes which were known to be associated with HPA were found. But two novel mutations in *DNAJC12* (NM_021800.2) which he inherited from each parent, c.306C>G (p.His102Gln) and c.182delA (p.Lys61Argfs*6), were found. The two novel variants of *DNAJC12* were predicted as damaging the function and had never been reported before in publicly available databases and local population databases. According to the ACMG standards and guidelines for the interpretation of sequence variants, these novel mutations are likely pathogenic or pathogenic (Table 2). Based on reported patients with HPA, who were diagnosed to be *DNAJC12* deficient, we finally identified HPA in the proband which resulted from the compound heterozygosity mutations in *DNAJC12*. Detailed history of the family and sequencing results is shown in Figure 1.

**TABLE 2:** Details of pathogenic identified variants of *DNAJC12*

Nucleotide change	Amino acid change	Variants Type	Exon Num	SIFT	PolyPhen−2	Mutation Taster	ACMG/AMP classification
c.182delA	p.Lys61Argfs*6	frameshift	3	NA	NA	disease causing	PVS1 + PM2+PP4 (P)
c.306C > G	p.His102Gln	missense	4	damaging	damaging	disease causing	PM2 + PM3+PP3 + PP4 (LP)

Note

SIFT classification: damaging, tolerated; PolyPhen‐2 classification: damaging, probably damaging, benign; Mutation Taster classification: disease causing, polymorphism; ACMG/AMP classification: PVS1: loss of function variant; PM2: absence of the variant in general population; PM3: for recessive disorders, detected in trans with a pathogenic variant; PP3: multiple lines of computational evidence support a deleterious effect; PP4: patient's phenotype specifically matches with the condition. P = pathogenic; LP = likely pathogenic. NA, not available. The variant was described by the accession number NM_021800.2 (ENSG00000108176) for *DNAJC12*.

### 3.3 Clinical management and follow‐up of the *DNAJC12*‐deficient patient

**FIGURE 2:**
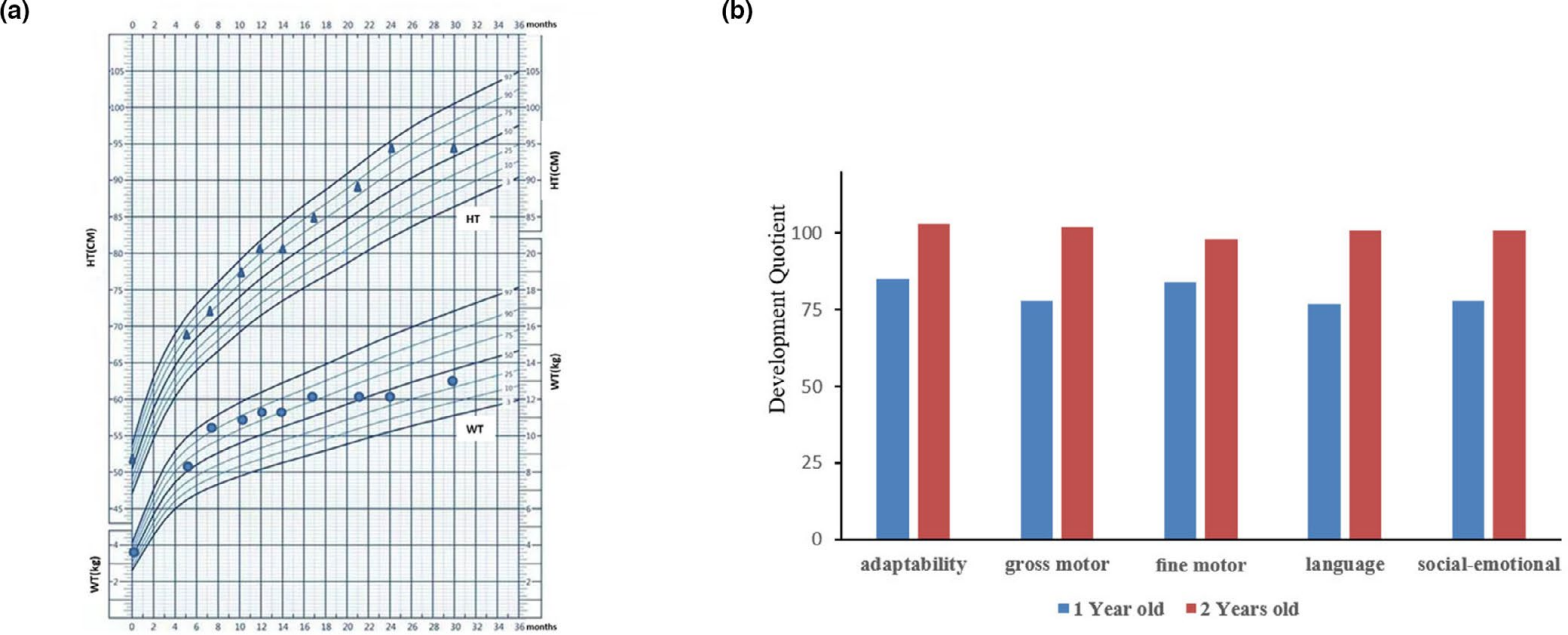
Growth curve and Gesell developmental assessment of the patient. (a) Growth curve of height and weight. The patient growth curve was referred to Height‐for‐age and weight‐for‐age Percentiles of Chinese boys (Capital Institute of Pediatrics., 2007). HT: height; WT: weight. Blue triangles represented HT of the patient. Blue dots represented WT of the patient. (b) Gesell developmental assessment of the patient. Development quotient (DQ) score was the parameter for evaluating development. DQ > 85: normal; 55 < DQ < 75: mild impairment; 40 < DQ < 55: moderate impairment; 25 < DQ < 40: severe impairment

The patient was placed on a phenylalanine‐restricted diet as he was affected by HPA. When he was 1‐year‐old, the Gesell developmental assessment of the patient on adaptability, gross motor, fine motor, language, and social‐emotional characteristics with the developmental quotients 85, 78, 84, 77, and 78, respectively, were performed. All the values for these parameters approached minimum normal reference values (Figure 2). After diagnosis of *DNAJC12* deficiency, the patient received a combined treatment of 5‐hydroxytryptamine (50 mg/day), levodopa (100 mg/day), and tetrahydrobiopterin (25 mg/day). The re‐examination of Gesell developmental assessment of the patient was normal at 2 years old, which was 103, 102, 98, 101, and 101 on adaptability, gross motor, fine motor, language, and social‐emotional characteristics with the developmental quotients, respectively (Figure 2). Through follow‐up till 30 months, the patient did not onset obviously developmental delay (Figure 2).

## 4 DISCUSSION

**FIGURE 3:**
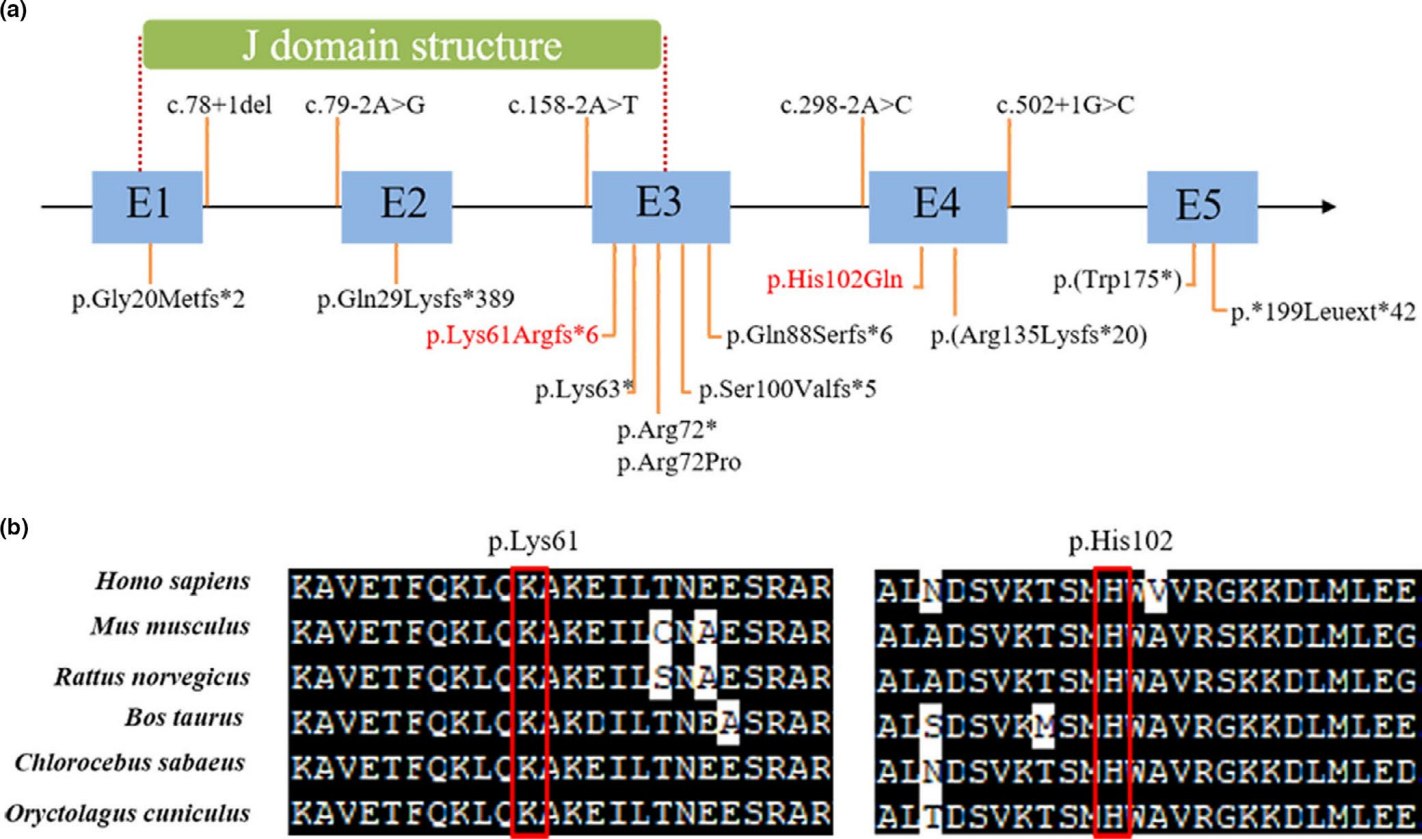
Summary of reported mutations and novel mutations in this study. (a)The mutations marked with red color are novel mutations in the present study. The blue box represents each exon. The green box represents J domain structure. The previously reported mutations refer to BIOPKU (http://www.biopku.org). (b) Sequence alignment of various mammalian species DNAJC12 proteins shows a conserved of two residues

*DNAJC12* is a member of the DnaJ/HSP40 family that has been designated as JDP1 and detected its expression in brain, heart, testis, kidney, and so on. Abnormal expression of *DNAJC12* in cancer tissues might be used as a potential marker for the disease (De Bessa et al., 2006). Recently, DNAJC12 has been demonstrated by interacting with several proteins, including tyrosine hydroxylase (TH), peripheral tryptophan hydroxylase 1 (TPH1), and neuronal TPH2 (Anikster et al., 2017). PAH enzyme activity is reduced in the presence of *DNAJC12* mutations (Anikster et al., 2017). *DNAJC12* interacts with the ATPase domain of Hsp70 proteins to stimulate ATP hydrolysis through the J domain structure which spans positions 14–79 in *DNAJC12* (Figure 3) (Kampinga & Craig, 2010). Also, it interacts with the aromatic amino acid hydroxylases (AAAHs) and plays important role in proper folding of PAH (Anikster et al., 2017; Jung‐Kc et al., 2019). The BIOPKU (http://www.biopku.org) has reported 15 mutations of *DNAJC12*, which mainly are genetic null mutations (nonsense, frameshift, splice‐site mutations) (Figure 3). The patient in the present study carried compound heterozygosity mutations of c.306C > G (p.His102Gln) and c.182delA (p.Lys61Argfs*6) in *DNAJC12*, which was inherited, respectively, from his parents who showed no symptoms of disease. The frameshift variant is located in the J domain structure, which was predicted to produce a truncated protein which would affect the normal function of *DNAJC12*. The missense variant had not been reported previously in publicly available databases, such as BIOPKU (http://www.biopku.org), ClinVar (https://www.ncbi.nlm.nih.gov/clinvar/), HGMD (http://www.hgmd.cf.ac.uk/ac/validate.php), EXAC (http://exac.broadinstitute.org/), and so on. Multiple alignment of the two residues in DNAJC12 proteins from various mammalian species showed conserved (Figure 3). Taken together, the compound heterozygosity mutations were considered to cause the HPA manifestations in the affected child.

As far as we know, this is the first report of patient with *DNAJC12* deficiency in Guangxi Zhuang Autonomous Region. Both the variants in our study have never been reported. According to our in‐house database (*n* = 2,757), the allele frequency is nearly 0.0002. Other reported patients with *DNAJC12* deficiency were rare in the eastern Asian population. Furthermore, most of the loss‐of‐function variants of *DNAJC12*, noted in the Exome Aggregation Consortium (ExAC) database (http://exac.broadinstitute.org/), are not found in the eastern Asian population and the allele frequency is extremely low. Therefore, we suppose that the incidence of *DNAJC12* associated with mild HPA is rare, especially in eastern Asian population.

Over the past decades, population newborn screening (NBS) has been shown to be critical for the early diagnosis and treatment of HPA (Hinton et al., 2016). The patient in our study was suspected of having HPA as a result of NBS. However, it should be noted that NBS results could be normal in some patients with *DNAJC12* deficiency (Anikster et al., 2017). Those patients would have intellectual disability and/or behavioral problems when they are first diagnosed. Therefore, this study would suggest that a more sensitive and convenient screening system for HPA should be established. The reported patients with *DNAJC12* deficiency usually receive BH4 with or without l‐dopa/carbidopa/5‐hydroxytryptophan treatment; as a result, BH4 supplementation not only supports in the aromatic amino acid hydroxylases (PAH, TH, and TPH) reactions, but also acts as a pharmacological chaperone that increases the stability of these enzymes similar to that occurs in BH4‐responsive PKU (Anikster et al., 2017; de Sain‐van der Velden, 2018). We also consider that the patient with *DNAJC12* deficiency should receive BH4 treatment which can be effective for correcting the biochemical abnormalities and long‐term prognosis. So early identification and clinical intervention of mild HPA can help prevent developmental delay and intellectual disability problems.

In this study, the main clinical symptom of the patient was HPA, but traditional experimental methods such as Sanger sequencing for several common genes could not provide the precise genetic diagnosis. Therefore, WES reanalysis is one of the important means for clinical diagnostic, especially for the patients have atypical clinical features. Furthermore, next‐generation sequencing is rapidly expanding in its use for screening and diagnosing diseases (Narravula & Garber, 2017). We anticipate that WES or WGS will fulfill their potential to determine new genes and minimize uncertain findings so that patients receive optimal clinical treatment for their condition.

## 5 CONCLUSIONS

In conclusion, we report on a patient with mild HPA and novel compound heterozygosity mutations in *DNAJC12* found by WES analysis, which was HPA without the presence of PKU and BH4 metabolic disorders. The patient benefited from early intervention of a restricted diet and effective treatment. Therefore, early diagnosis and treatment are important in order to improve the neurodevelopmental outcome of HPA patients.

## CONFLICTS OF INTEREST

The authors have no conflict of interest to disclose.

## AUTHOR CONTRIBUTIONS

Collection of clinical data: Xin Fan: Data analyzed and interpreted: Mengting Li, Qi Yang, Sheng Yi, Zailong Qin, and Jingsi Luo; Writing and review of original draft of the manuscript: Mengting Li and Xin Fan.

## Supporting information

Supplementary Material

## Data Availability

The data that support the findings of this study are available from the corresponding author upon reasonable request.
