# Supplementary material for: Two novel mutations in DNAJC12 identified by whole‐exome sequencing in a patient with mild hyperphenylalaninemia
Source: Mol Genet Genomic Med. 2020 Jun 10;8(8):e1303. doi: 10.1002/mgg3.1303 (PMC7434608; doi:10.1002/mgg3.1303)
Supplement: Supplementary file 1 — Supplementary Material [file MGG3-8-e1303-s001.docx]

Table S1. PCR primers and conditions used for Sanger sequencing

| Exon | Sequence (5′-3′) | | Product size(bp) | Annealing temperature(°C) |
| --- | --- | --- | --- | --- |
|  | Forword | Reverse |  |  |
| 3 | GCCGAGCACTTTACATCCA | TGGTTTTCTCCCTCCTATCG | 440 | 60 |
| 4 | GGTACAAAAGGGTGTATGGTGAA | CAAGGTTTCCCTTCTGAGGA | 604 | 60 |
